# Immunomodulatory Potential of Quinoline Q3, a Selective Inhibitor of the Canonical NF‐κB Pathway in Macrophages

**DOI:** 10.1002/cmdc.70370

**Published:** 2026-07-06

**Authors:** Konstantinos Michail, Panagiotis Ntavaroukas, Vasiliki Petriki, Evangelos Tsioupros, Stella Manta, Barry J. Campbell, Stamatia Papoutsopoulou

**Affiliations:** ^1^ Department of Biochemistry and Biotechnology University of Thessaly Larissa Greece; ^2^ Laboratory of Organic Chemistry Faculty of Chemistry Aristotle University of Thessaloniki Thessaloniki Greece; ^3^ Department of Infection Biology & Microbiomes Institute of Infection Veterinary and Ecological Sciences University of Liverpool Liverpool UK

**Keywords:** immune cells, inflammation, macrophage, NF‐κB, quinoline, transcription

## Abstract

The NF‐κB transcription factor pathway is a major driver of inflammation, regulating proinflammatory cytokine synthesis in innate immune cells. Its dysregulation is linked to chronic inflammatory diseases, as well as autoimmune conditions. We investigated the inhibitory effect of a novel quinoline Q3 on NF‐κB activation in murine and human macrophages. Without little/no effect on cell survival or proliferation of J774A.1 macrophages, lipopolysaccharide (LPS)‐induced NF‐κB‐regulated luciferase signal in lentivirally transduced macrophages was significantly reduced in the presence of 5 µM Q3. Q3 also inhibited LPS‐induced transcription of proinflammatory genes *Tnf*, *Il6*, and *Il12b* as shown by qPCR. Q3 was also seen to significantly reduce intracellular levels of pre‐TNF; however, Q3 could not diminish the released cytokines, in both mouse and human macrophages. NF‐κB ELISA further revealed that Q3 inhibited p65 DNA‐binding activity, while it enhanced p50 DNA‐binding, a combination that could lead to inhibition of target gene transcription, since in silico analysis revealed interaction between Q3 and the p50 homodimer/DNA complex. Q3 also inhibited p65/NF‐κB DNA‐binding in LPS‐stimulated human macrophages, but there was no impact of Q3 on c‐Rel DNA‐binding activity. Therefore, Q3 differentially interferes with the DNA‐binding of specific NF‐κB dimers, and this could be exploited to target NF‐κB‐regulated inflammation.

## Graphical abstract

The quinolone Q3 specifically enhances the DNA binding of the p50/p50 NF‐κB dimers, while inhibiting the activity of the p65 transcriptional activator, resulting in inhibition of the NF‐κB canonical pathway in lipopolysaccharide (LPS)‐stimulated macrophages.

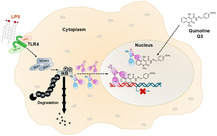

## 1 Introduction

Innate immune cells, such as macrophages, play a pivotal role as the body's first line of defense. They can detect pathogens, foreign material, and abnormal/damaged cells, and they can initiate inflammatory responses including activation of lymphocytes, the main players of the adaptive immune system [1]. Macrophages are also necessary for the resolution of inflammatory responses and in tissue homeostasis by dampening inflammation, clearing dead and dying cells, and promoting repair of mucosal damage [2]. At the cellular level, and during the initial phase of an inflammatory response, a macrophage utilizes mechanisms and molecules that are immediately available, such as the nuclear factor‐kappa B (NF‐κB) transcription factor pathway and therefore regulates within minutes the transcriptional response that will effectively guide the cell function and fate [3, 4]. NF‐κB represents a family of five transcription factors, namely, p50, p65(RelA), c‐Rel, p52, and Rel‐B, that form homo‐ and heterodimers, which upon activation translocate to the nucleus to regulate gene transcription. The dimers that are formed by the p50, p65, and c‐Rel subunits participate in the canonical (or classical) pathway [5]. They regulate inflammation by controlling, in cooperation with a variety of transcription factors, the expression of pro‐ and anti‐inflammatory cytokines, chemokines [6, 7, 8], nitric oxide [9], and processes such as phagocytosis [10]. Dysregulated canonical NF‐κB pathway activity causes chronic inflammatory diseases, such as asthma, diabetes, inflammatory bowel disease, inflammation‐associated cancer [11, 12], rheumatoid arthritis, and other autoinflammatory conditions [13, 14, 15]. Therefore, research has been focused on the field of potential therapeutic application of inhibition of NF‐κB signaling [3, 16, 17, 18].

Quinolines, the class of nitrogen‐containing heterocyclic aromatic compounds, are widely used as scaffolds in medicinal chemistry [19]. Quinolines, and related quinolones (quinolines with a ketone (oxo) group), have been used as antimicrobial agents because of their action against a variety of microorganisms, such as bacteria, fungi, viruses, and parasites [20]. These compounds usually inhibit microbial DNA synthesis by targeting enzymes such as DNA gyrases and topoisomerases [21] but have been reported to inhibit NF‐κB activation, mainly in in vitro models [22]. Quinolones have been shown to inhibit microglia inflammatory responses mediated by NF‐κB signaling downstream of Toll‐like receptor 4 (TLR4) [23], as well as inhibiting macrophage chemotaxis [24]. The anti‐inflammatory activity of 8‐(tosylamino) quinoline in lipopolysaccharide (LPS)‐stimulated RAW264.7 cells and peritoneal macrophages occurs through inhibition of Akt/NF‐κB signaling [25]. Similarly, in human peripheral blood monocytes infected with *Aspergillus fumigatus*, the fluroquinolone moxifloxacin was shown to inactivate the p65‐NF‐κB signaling pathway, mitogen‐activated protein kinases (MAPKs) ERK1/2 and p38, and proinflammatory cytokine synthesis [26]. We recently reported a novel quinoline molecule, quinoline Q3 (4‐hydroxy‐*N*′‐(4‐methoxybenzylidene)‐1‐methyl‐2‐oxo‐1,2‐dihydroquinoline‐3‐carbohydrazide), synthesized from a well‐known quinoline ethyl 4‐hydroxy‐1‐methyl‐2‐oxo‐1,2‐dihydroquinoline‐3‐carboxylate (Q1) [27]. The novel quinoline Q3 compound suppressed the canonical NF‐κB pathway in tumor necrosis factor (TNF)‐activated epithelial cell‐lines, such as HeLa, Intestine‐407, DLD‐1, and HT‐29, likely through interference with the DNA‐binding activity of the transcription factor [27]**.**

Here, we evaluated the potential for the quinoline Q3 to interfere with the canonical NF‐κB pathway in bacterial PS‐triggered innate immune cells, including the J774.A1 murine macrophage cell‐line and in human primary macrophages. In silico analyses revealed that Q3 preferentially interacts with the p50/DNA complex. We showed that Q3 promotes LPS‐induced p50/NF‐κB DNA‐binding, whereas it inhibits p65/NF‐κB activation in both murine and human macrophages and has no impact on c‐Rel//NF‐κB activity. We also showed that Q3 differentially interfered in the transcription of different proinflammatory cytokines with maximum effect on the expression of ll12b, a result that could be exploited to target NF‐κB‐regulated inflammation.

## 2 Results and Discussion

### 2.1 Quinoline Q3 Does Not Impact on J774A.1 Macrophage Viability

**FIGURE 1:**
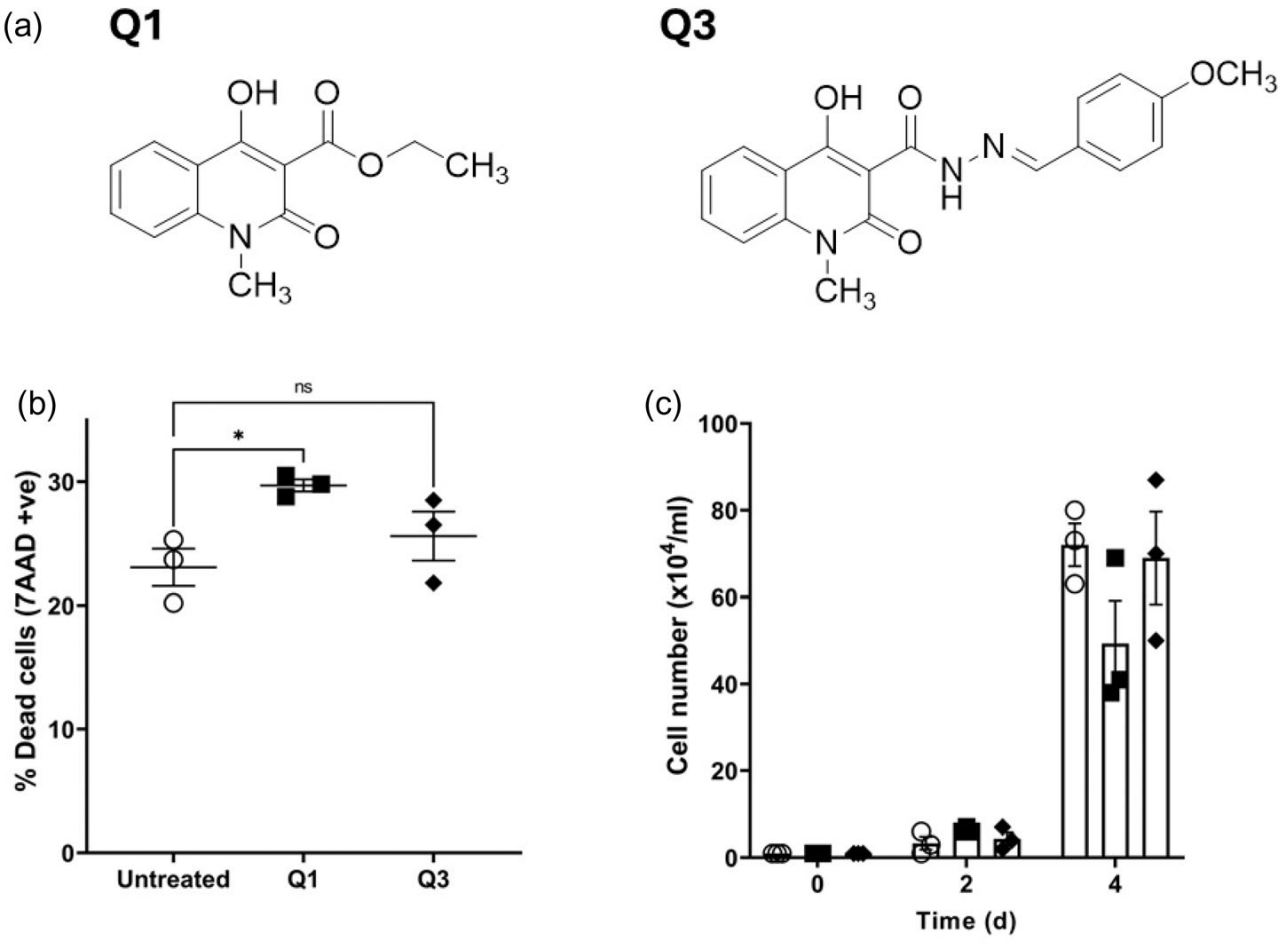
Effect of quinolines Q1 and Q3 on survival and proliferation of murine J774A.1 macrophages. Macrophage cultures were left untreated (open circles) or treated with 5 µM Q1 (black squares) or Q3 (black diamonds) for 24 h (*n* = 3). (a) Chemical structures of Q1 and Q3 compounds, (b) cells were harvested and stained with 7AAD to support cell viability analysis by flow cytometry, with data presented as % dead cells, or (c) trypan blue to measure live cells per mL. One‐way ANOVA was performed, followed by Tukey's post hoc multiple comparisons of treatment means. Significant differences, *p < 0.05 compared to DMSO‐treated controls, ns, nonsignificant. Data representative of at least two independent experiments.

In experiments where J774A.1 macrophages were either left untreated or were incubated in the presence of 5 µM Q3 for a time course up to 24 h, results from 7AAD flow cytometry analysis showed that Q3 did not cause any significant cell death throughout the duration of the 24 h experiment (Figure 1a). Conversely, treatment with the unmodified quinoline Q1, from which Q3 was derived, could induce cell death (*p* < 0.05; ANOVA, *n* = 3). Q1 was not used in further experiments. A similar experiment was performed where macrophage cultures were incubated for a longer period, for up to 4 days, with cell numbers determined using Trypan blue staining. Again, no statistically significant effect on cell proliferation was observed in the presence of Q3 (Figure 1b).

### 2.2 Quinoline Q3 Inhibits the NF‐κB Canonical Pathway in Murine and Human Macrophages

**FIGURE 2:**
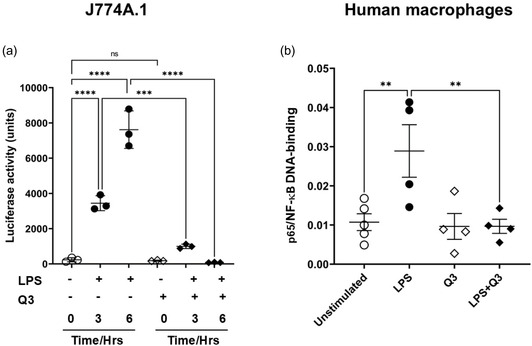
The quinoline Q3 inhibits LPS‐induced NF‐κB activation in murine and human macrophages. (a) NF‐κB‐regulated luciferase activity in lentiviral construct κB‐NLSluc transduced J774A.1 murine macrophages either left unstimulated or stimulated with 100 ng/mL LPS for 3 or 6 h in the absence or presence of 5 µM Q3 (*n* = 3 for each treatment group). Luminescence activity was detected in an Infinite F200 plate reader. (b) p65/NF‐κB ELISA in human PBMC‐derived macrophages either left unstimulated or stimulated with 100 ng/mL LPS for 1 h (*N* = 4–5). One‐way ANOVA was performed followed by Tukey's post hoc multiple comparisons of treatment means. Significant differences, **p < 0.01, ***p < 0.001, ****p < 0.0001. Data representative of at least two independent experiments.

To evaluate the effect of Q3 on NF‐κB activation, a lentiviral transduction approach was used where murine J774A.1 macrophage cultures were transduced with a reporter construct that expresses luciferase under the canonical pathway NF‐κB promoter, as previously described [28]. Cells were stimulated with LPS for a time course up to 6 h in the absence or presence of Q3, and luciferase signal was measured. LPS (100 ng/mL) significantly induced luciferase activity at both 3 and 6 h (14.5‐fold and 32‐fold, respectively, *p* < 0.0001); see Figure 2a. The LPS‐induced signal was significantly reduced at 3 h (3.5‐fold, *p* < 0.001), and completed abrogated at 6 h, in the presence of 5 µM Q3 (*p* < 0.0001; ANOVA, *n* = 3). We further evaluated the effect of Q3 on human peripheral blood mononuclear cell (PBMC)‐derived macrophages obtained from healthy volunteers, either left unstimulated or were treated with 100 ng/mL LPS in the absence or presence of Q3 for 1 h. RIPA total cell lysates used for measurement of p65/NF‐κB DNA‐binding activity showed that p65 activity was dramatically reduced in the presence of the quinoline Q3 inhibitor (Figure 2b), confirming that this compound exerts its inhibitory function in both murine and human macrophages.

### 2.3 NF‐κB‐Dependent Transcription and Intracellular TNF Levels Are Affected by Quinoline Q3

**FIGURE 3:**
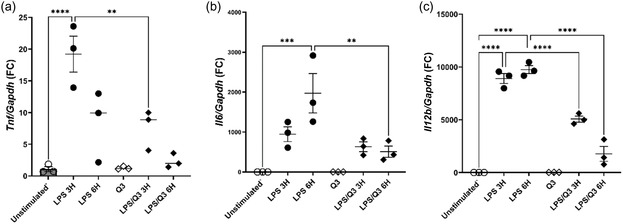
Effect of quinoline Q3 on LPS‐induced cytokine gene expression in murine J774.A1 macrophages. Total RNA was isolated from cells either left unstimulated or stimulated with 100 ng/mL LPS for 3 or 6 h in the absence or presence of 5 μM quinoline Q3. Following total RNA isolation and reverse transcription, quantification of mRNA abundance was performed by qPCR for (a) *Tnf*, (b) *Il6*, and (c) *Il12b*. Data are expressed as fold change (FC) relative to expression of housekeeping gene *Gapdh*. One‐way ANOVA was performed, followed by Tukey's post hoc multiple comparisons of treatment means. Significant differences, **p < 0.01 and ****p < 0.0001; ANOVA, *n* = 3.

In macrophages, Toll‐like receptor signaling and downstream activation of the canonical NF‐κB pathway lead to proinflammatory cytokine synthesis, e.g., TNF, IL‐6, and IL‐12 [6]. The regulation of these genes is complex, and studies have shown that there is plasticity and NF‐κB dimer‐specific regulation of the target genes [29]. We therefore assessed whether the Q3 compound had differential impact on the expression of the *Tnf*, *Il6*, and *Il12b* genes. J774A.1 macrophages were cultured and stimulated in the absence or presence of 5 μM Q3 for up to 6 h. Q3 inhibited the transcription of *Tnf* by 60% at 3 h, *Il6* by 75% at 6 h (Figure 3a), and *Il12b* by 43% at 3 h (Figure 3b) and by 82% at 6 h (Figure 3c); all *p* < 0.01, ANOVA (*n* = 3).

**FIGURE 4:**
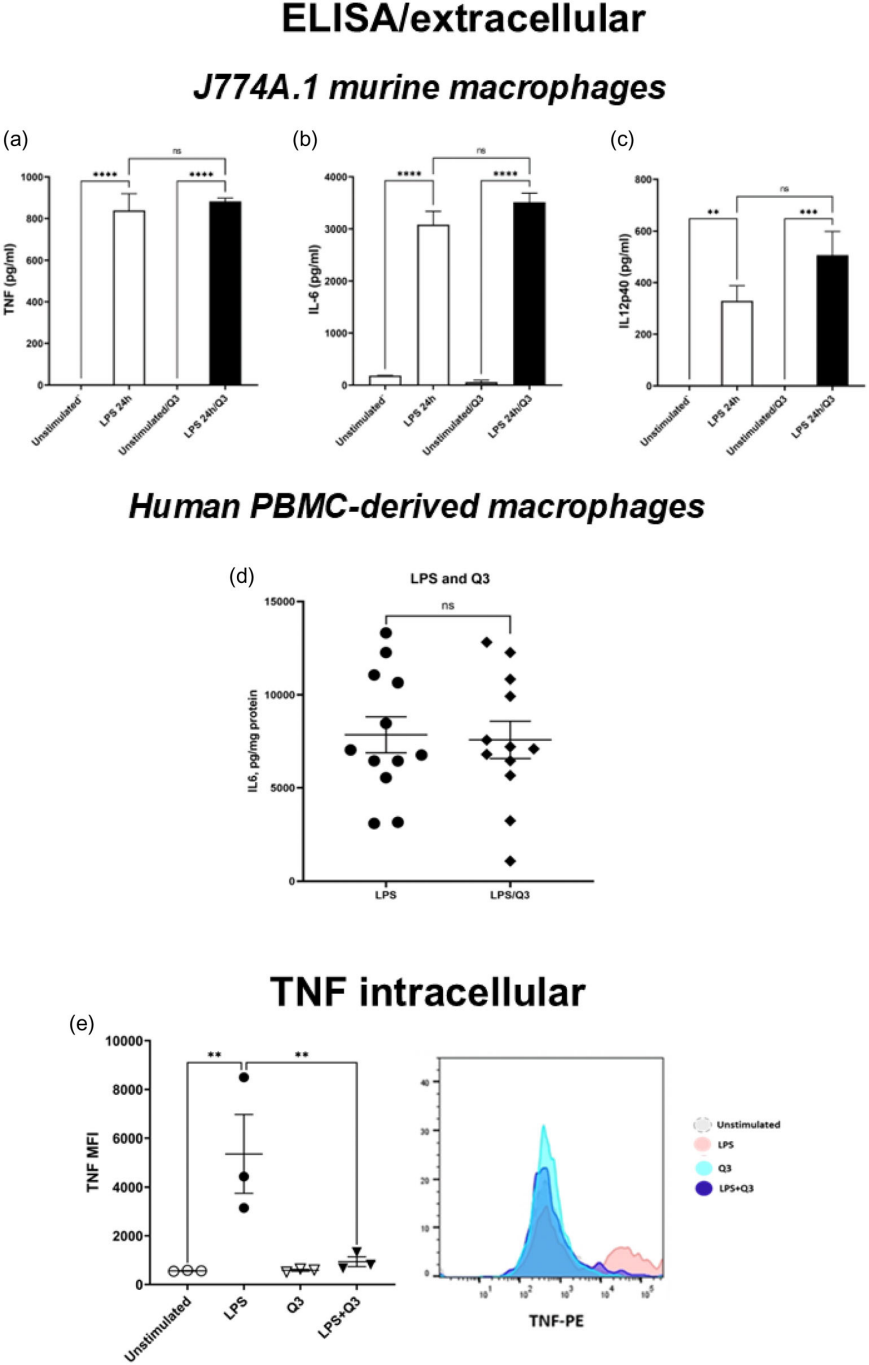
Quinoline Q3 effect on LPS‐induced proinflammatory cytokines TNF, IL‐6, and IL‐12p40 from murine J774A1 macrophages. Cells either left unstimulated or stimulated with 100 ng/mL LPS for 24 h in the absence or presence of 5 µM Q3. Quantification of cytokine levels released to culture medium was performed by ELISA specific for murine (a) TNF, (b) IL‐6, and (c) IL‐12p40. (d) For intracellular staining of pre‐TNF, J774A.1 macrophages were either left unstimulated or stimulated with 100 ng/mL LPS for 2 h in the absence or presence of 5 μM Q3 and in the presence of 1 μg/mL Brefeldin A (*n* = 3). Left‐hand panel: flow cytometry data presented as mean fluorescence intensity (MFI) from *n* = 3 experiments. Right‐hand panel: representative experimental dataset; unstimulated cells (gray), Q3 treated alone (cyan), LPS stimulated (pink), and LPS stimulation in the presence of Q3 (blue). (e) Human PBMC‐derived macrophages (*N* = 6, *n* = 2) were stimulated with 100 ng/ml LPS for 24 h in the absence or presence of 5 µM Q3. Quantification of cytokine levels released to culture medium was performed by ELISA specific for human IL‐6. One‐way ANOVA was performed, followed by Tukey's post hoc multiple comparisons of treatment means. Significant differences, ** *p* < 0.01, ***p < 0.001, and ****p < 0.0001; ns, nonsignificant.

Next, we measured the levels of the secreted cytokines, soluble TNF, IL‐6, and IL‐12p40, released to the culture medium following stimulation for 24 h with LPS, as detected by ELISA. LPS induced the secretion of all three proinflammatory cytokines examined, but no statistically significant differences were detected in chosen time points and between untreated samples or those treated with Q3 (Figure 4a–c). In a similar experiment using human PBMC‐derived macrophages from six healthy donors, we could not detect statistically significant differences at IL‐6 levels released from LPS‐activated macrophages 24 h post‐LPS addition (Figure 4d).

It is known that TNF is initially synthesized as pre‐TNF and upon maturation the soluble form is released in the extracellular space [30]. Therefore, we examined the pre‐TNF cytoplasmic levels by intracellular flow cytometry based on our previous study [31]. LPS induced the synthesis of pre‐TNF, as expected, and the presence of Q3 inhibited its synthesis (Figure 4e), a result that reflects the transcriptional analysis.

### 2.4 Quinoline Q3 Differentially Affects NF‐κB Subunit DNA‐Binding Activity

**FIGURE 5:**
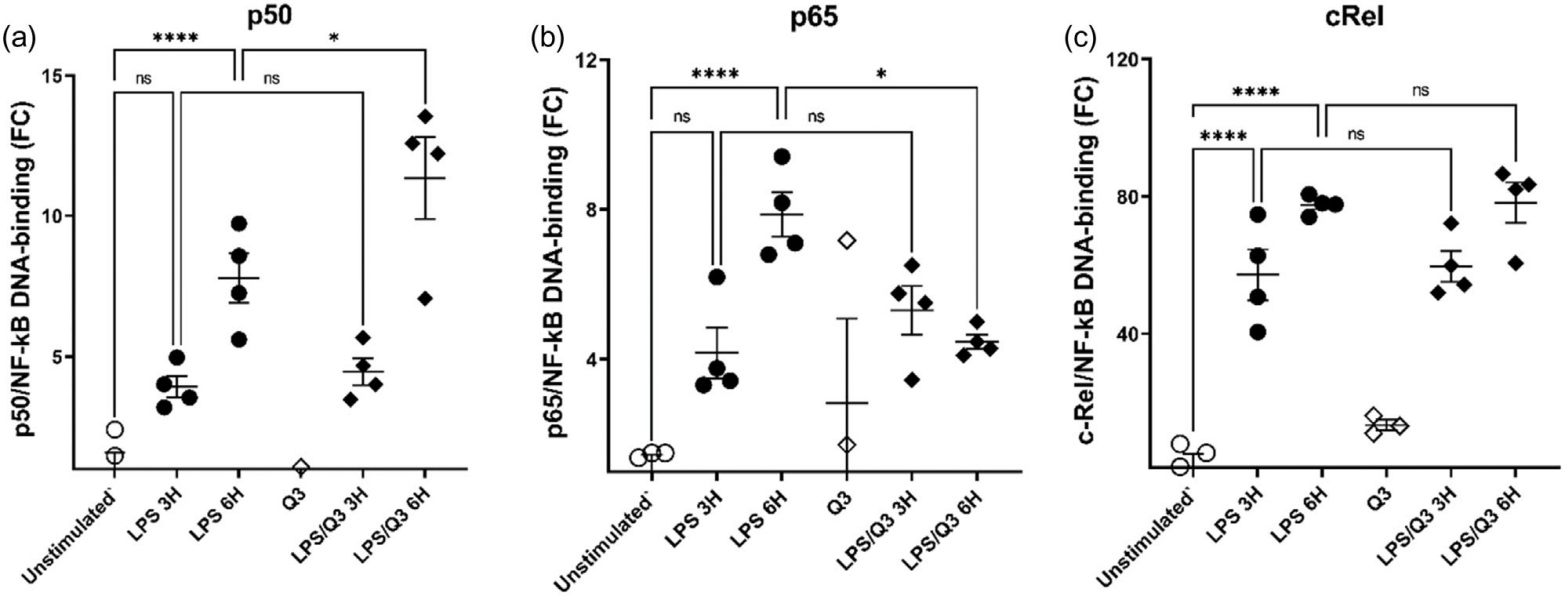
Quinoline Q3 differentially affects the NF‐κB subunit DNA‐binding activity. Cells either left unstimulated or stimulated with 100 ng/mL LPS for 3 and 6 h in the absence or presence of 5 µM Q3 (*n* = 3–4). NF‐κB ELISA was used to quantify the DNA‐binding activity of subunit (a) p50, (b) p65, and (c) c‐Rel, with the absorbance normalized against total protein levels. Data presented as fold change (FC) compared to unstimulated controls. One‐way ANOVA was performed, followed by Tukey's post hoc multiple comparisons of treatment means. Significant differences, *p < 0.05 and ****p < 0.0001; ns, nonsignificant.

We also set to examine to what extent the Q3 compound affects the activity of each of the NF‐κB subunits that participate in the canonical pathway and regulate inflammatory responses. For this, J77A4.1 macrophages were cultured and stimulated with 100 ng/mL LPS in the absence or presence of 5 μM Q3 for 3 and 6 h. Total RIPA lysates were used to measure NF‐κB‐DNA binding activity for the p50, p65, and c‐Rel subunits. The quinoline Q3 had a positive effect on p50 activity by 45% (*p* < 0.05, ANOVA; *n* = 3) at the latest point and it significantly inhibited by 50% the p65 DNA‐binding at 6 h (*p* < 0.05); see Figure 5. In contrast, there was no effect on c‐Rel activity at any time point.

### 2.5 In Silico Analysis Reveals Interaction between Q3 and the P50 Homodimer/DNA Complex

**FIGURE 6:**
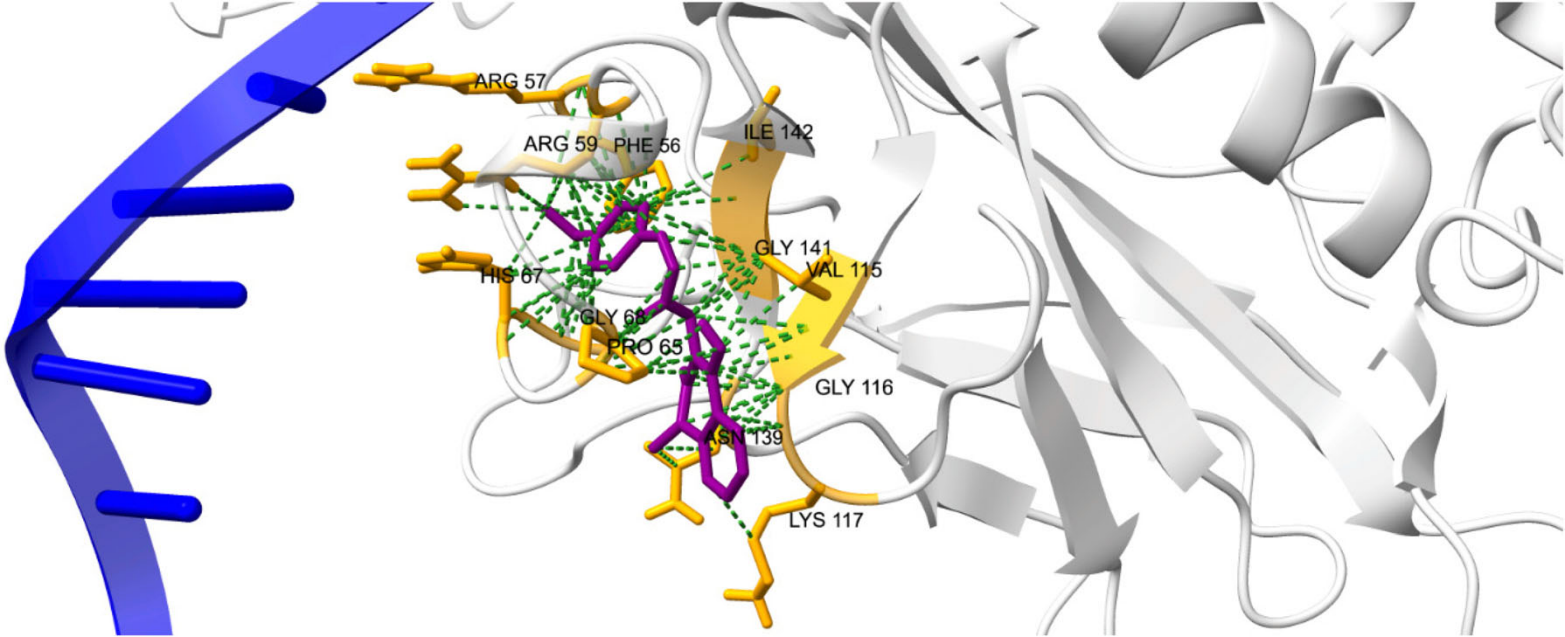
The 12 amino acid residues define a putative pocket (yellow) for Q3 during p50‐mediated DNA binding. DNA is shown in blue and Q3, in purple, is seen forming multiple contacts with the pocket residues (as shown by green dotted lines), which may explain the low binding energy observed in the docking experiments. (Figure was created using the ChimeraX software v.1.11.1) [32, 33].

In a previous study, using an in silico analysis, we showed that quinoline Q3 potentially interfered with the DNA‐binding activity of the p65/NF‐κB subunit [10]. We therefore examined whether Q3 could interact with the p50/NF‐κB subunit. Analysis revealed Q3 showing more favorable binding to p50/p50 homodimer in the presence of DNA (Table 1). Q1 was utilized as a control, which showed similar binding to p50 in the absence or presence of DNA. As shown in Figure 6, Q3 interacts with a number of residues (Arg 59, His 67, Phe 56, Gly 116, Val 115, Arg 57, Gly 141, Pro 65, Gly 68, Ile 142, Asn 139, Lys 117) that belong in the p50 Rel homology domain (RHD) and they form a pocket of approximately the size of 4 Å. No interactions of Q3 with the DNA itself were identified, probably because Q3 seems to bind very tightly in the pocket defined by these residues and therefore affecting the p50/p50 DNA binding by triggering conformational changes.

**TABLE 1:** Binding energies (kcal/mol) of Q1 and Q3 with p50 homodimer and p50 homodimer–DNA complex.

Q1 + p50	Q3 + p50	Q1 + p50/DNA	Q3 + p50/DNA
−7.18	−6.75	−7.13	−7.82
−6.69	−6.71	−6.63	−7.57
−6.67	−5.71	−6.57	−6.68
−6.53	−5.69	−6.31	−5.77
−6.26	−5.65	−6.14	−5.61
−6.12	−5.41	−6.06	−5.55
−5.76	−5.13	−5.52	−5.26
−5.73	−4.71	−5.38	−5.08
−5.1	−4.6	−4.76	−4.75
−4.97	−4.57	−4.56	−4.05

*Note:* The Lamarckian algorithm was used for setting in silico molecular docking parameters, where the top 10 binding energies were retrieved for each ligand, indicating the strongest molecular interactions.

## 3 Conclusion

The novel quinoline compound Q3 interferes with the canonical NF‐κB signaling pathway downstream of TLR‐4 in both J774.A1 murine macrophage cell‐line and in human primary macrophages. In silico studies revealed that Q3 preferentially binds to p50 homodimer/DNA complex and promotes p50 DNA‐binding. On the other hand, Q3 also inhibits p65/RelA activity. Both these actions of Q3 likely lead to reduction of transcription. Moreover, Q3 was shown to have no impact on c‐Rel NF‐κB subunit activity as revealed by transcript quantification of specific cytokine target genes. At the protein level, intracellular pre‐TNF was dramatically reduced in the presence of Q3, but the extracellular release of cytokines induced by LPS (for up to 24 h) was not affected, possibly due to autocrine/paracrine complex regulatory network/interaction pathways. Interestingly, the selectivity that Q3 shows in its interactions with the NF‐κB subunits could be further exploited to target specific NF‐κB‐regulated processes.

## 4 Materials and Methods

### 4.1 Cell Culture—Human Cells

The J774.A1 murine macrophage cell‐line was obtained from the European Collection of Animal Cell Culture (ECACC Cat# 91051511, RRID:CVCL_0358; Porton Down, Salisbury, UK) and routinely maintained in Roswell Park Memorial Institute (RPMI) 1640 medium, supplemented with 10% (v/v) fetal bovine serum (FBS), 100 U/mL penicillin, 100 µg/mL streptomycin, 2 mM L‐glutamine (all Sigma–Aldrich, Poole, UK), 1 mM sodium pyruvate, and 10 mM HEPES pH 7.0 (Gibco, Paisley, UK) in a humidified incubator at 37°C, in an atmosphere of 5% CO_2_. For passaging, adherent cells were detached using a sterile cell scraper, followed by gentle aspiration with a pipette to a cell suspension and counted using either a Neubauer cell counting chamber (Sigma–Aldrich) or a TC10 automated cell counter (Bio‐Rad, Hemel Hempstead, UK). Cells in suspension were then reseeded to flasks, either for maintenance or to plates for experimentation. Human PBMCs were obtained from healthy volunteer blood taken following informed consent, differentiated to macrophages with 50 ng/mL human macrophage colony‐stimulating factor (PreproTech EC Ltd., London, UK) and cultured as previously described [34]. LPS isolated from adherent, invasive *E. coli* LF82 was used for macrophage TLR4 activation, as per previous studies [12, 13]. The novel inhibitor of the canonical NF‐κB transcription factor pathway, quinoline Q3 (4‐hydroxy‐*N*′‐(4‐methoxybenzylidene)‐1‐methyl‐2‐oxo‐1,2‐dihydroquinoline‐3‐carbohydrazide), and its parent compound Q1 (ethyl 4‐hydroxy‐1‐methyl‐2‐oxo‐1,2‐dihydroquinoline‐3‐carboxylate) were synthesized and used as described previously [27].

Ethical approval for the studies using human peripheral blood‐derived mononuclear cells reported here was obtained from the Ethics Committee for Research of University of Thessaly (54/05.06.2025), with all experiments performed under the supervision of S. Papoutsopoulou. Informed, written consent was obtained from the healthy adult participants.

### 4.2 Lentivirus Production and Lentiviral NF‐κB Transcriptional Activity Reporter Assay

p65/NF‐κB transcriptional activity was monitored using the lentiviral construct κB‐NLSluc that expresses luciferase under the control of the classical NF‐κB promoter, as described previously [28]. Following transfection of HEK293T cells obtained from the American Type Culture Collection (ATCC Cat# CRL‐3216, RRID:CVCL_0063 ‐ LGC Standards, Teddington, UK) with packaging and target transfer vectors, cell culture medium from one well of a 6‐well plate was passed through a 70 μm cell strainer (BD Biosciences, Heidelberg, Germany) and the virus was precipitated using PEG‐it Virus Precipitation Solution (System Biosciences, Palo Alto CA, USA) as per manufacturer instructions. The virus was then resuspended in 100 mL medium and used fresh to infect the target cells. J77A4.1 macrophages (at 1 × 10^4^ cells per well) were seeded to 96‐well plates (Greiner CELLSTAR flat bottom; Sigma–Aldrich) in 100 μL medium. Following culture overnight, the cells were either left untreated or transduced with NF‐κB/luc lentivirus in the presence of TransDux Virus Transduction Reagent (System Biosciences) in a final volume of 200 μL. The medium was replaced 24 h later, and cultures were incubated for a further 3 days. On the day of the experiment, cultures were either pretreated with the quinoline Q3 or dimethyl sulfoxide (DMSO) vehicle for 30 min prior to stimulation with LPS (100 ng/mL) in a final volume of 200 μL. At the end of the period of stimulation, medium was removed, cells were washed once with phosphate‐buffered saline (PBS) pH 7.3 (Gibco) and luciferase activity was measured following cell lysis using a Bright‐Glo kit (E2620 – Promega, Southampton, UK), as per manufacturer instructions. Cell lysates were transferred to 96‐well white wall plates (Nunc‐Immuno microwell P8616; Sigma–Aldrich) and luminescence was detected in an Infinite F200 plate reader (Tecan, Reading, UK).

### 4.3 Cell Viability Assays

J77A4.1 macrophages were cultured in 12‐well culture plates (Greiner CELLSTAR; Sigma–Aldrich) at 0.5 × 10^6^ cells/well in 2 mL medium and incubated for the time course required. At each time point the cells were washed with cold PBS, manually scraped, gently aspirated by pipetting, and resuspended in 100 µL PBS. Cells were stained with Invitrogen 7‐amino actinomycin D (7AAD) according to manufacturer guidelines (Fisher Scientific: Loughborough, UK) and analyzed by flow cytometry using a Cytomics FC 500 Beckman Coulter flow cytometer (Beckman Coulter, Inc., Fullerton CA, USA). Raw data analysis was performed using the CXP software (Beckman Coulter). For Trypan blue staining, the cells were washed with cold PBS, manually scraped, and resuspended in 100 µL PBS. Equal volume of filtered 0.4% (v/v) trypan blue (Gibco) was added and the number of dead cells taking up the blue dye compared to those impermeable to the dye (live) cells was visualized under a microscope and enumerated using a Neubauer cell counting chamber.

### 4.4 Enzyme‐Linked Immunosorbent Assays

Enzyme‐linked immunosorbent assay (ELISA) was used to measure the levels of proinflammatory cytokines released from J77A4.1 macrophages to cell culture medium in response to treatment with 100 ng/mL LPS over 24 h, including TNF and interleukins IL‐6 and IL‐12 p40. The murine TNF (#900‐T54) and IL‐6 (#900‐K50) ELISA kits were sourced from PreproTech EC Ltd. (London, UK), and the Murine IL‐12/IL‐23 p40 Allele‐specific DuoSet ELISA [DY499] was obtained from Bio‐Techne Ltd (Abingdon, UK). The Invitrogen human IL‐6 uncoated ELISA kit (#88‐706‐88) was sourced from Life Technologies Ltd. (Paisley, Scotland). For ELISA of NF‐κB activity, total cell lysates were prepared in radioimmunoprecipitation assay (RIPA) buffer (Sigma–Aldrich). Lysates were then assayed in duplicate, using a TransAm NF‐κB family member kit to measure activity of p50, p65, and c‐Rel (Active Motif, Waterloo, Belgium). Given that the c‐Rel antibody within this kit can only be used with human samples, to assay for murine c‐Rel activity, an anti‐c‐Rel rabbit polyclonal antibody (sc‐71; Santa Cruz Biotechnology, Inc., Heidelberg, Germany) was substituted as previously performed [35]. Data were normalized against total protein measured using a Pierce BCA Protein Assay Kit (Fisher Scientific, Loughborough, UK).

### 4.5 RNA Extraction and qPCR

RNA extraction and purification from cells was performed using the RNeasy mini kit (Qiagen, Manchester, UK). Purified RNA were reverse transcribed using the high‐capacity RNA‐to‐cDNA kit (Applied Biosystems, Paisley, UK), and cDNA stored at −20°C. Real‐time quantitative PCR (qPCR) was performed using 50 ng total cDNA in 96‐well plates (Roche, Burgess Hill, UK) with Taqman Fast advanced master mix (Applied Biosystems) and Taqman Gene Expression Assay probes (Applied Biosystems), using a qPCR LightCycler 480 (Roche). Taqman Gene Expression Assay probes used were *Tnf* (Mm00443258_m1), *Il6* (Mm00446190_m1), and *IL12b* (Mm01288989_m1) and results normalized to *Gapdh* (Mm99999915_g1). Conditions for qPCR were as follows: one cycle of: 120 s at 50°C, 20 s at 95°C; 40 cycles of 3 s at 95°C, 30 s at 60°C, and 20 s at 60°C; one cycle at 120 s at 72°C and 30 s at 60°C, as described previously [36]. Cp values were calculated from second derivative analysis and relative quantification was calculated using 2^−ΔΔCt^ method [37].

### 4.6 Intracellular Flow Cytometry Staining for Pre‐TNF

J774A.1 cells were seeded at 0.5 × 10^6^ cells/well in a 6‐well plate in 2 mL medium and cultured overnight. The next day the cultures were either left untreated or treated with 5 µM Q3 for 30 min and then they were stimulated with 100 ng/mL LPS in the presence of a protein transport inhibitor Brefeldin A (BioLegend, London, UK) at 1 µg/mL, for 2 h. Culture medium was removed, cells were washed with 1 mL PBS/1% (v/v) FBS, detached by cell scraper, transferred to a new Eppendorf tube, and centrifuged at 400× *g* for 3 min. The cell pellet was resuspended and fixed in 0.5 mL Intracellular Fixation Buffer (00‐8333; Invitrogen) for 15 min at 4°C. The cells were rinsed with 1 mL PBS/1% (v/v) FBS and were permeabilized with 0.5 mL 70% (v/v) ice‐cold ethanol in deionized water for 20 min at 4°C. Cells were then washed with 1 mL PBS/1% (v/v) FBS and centrifuged at 400× *g* for 3 min. For intracellular TNF staining, cells were resuspended in 100 µL PBS/1% (v/v) FBS containing antimouse TNF‐PE conjugated antibody (Clone MP6‐XT22; BioLegend) and incubated for 30 min at room temperature in the dark. Cells were then washed twice with PBS/1% (v/v) FBS, resuspended in 0.5 mL PBS/1% (v/v) FBS, and subsequently analyzed on flow cytometry. Raw data analysis was performed using the CXP (Beckman Coulter) and FlowJo v10.8 Software (BD Biosciences).

### 4.7 In Silico Molecular Docking to Predict Q3 Interactions with NF‐κB p50/p50 Homodimers

In this study, the docking software AutoDock 4.2 was used [38] to perform blind docking analysis of the human NF‐κB p50 homodimer to assess potential Q3 binding sites. The Protein Data Bank ID of the crystal structure used is 1SVC (PDB ID: 1SVC; www.rcsb.org/, accessed on 6 March 2025). The experimental procedure was performed as per AutoDock documentation (https://autodock.scripps.edu/documentation/documentation/, accessed on March 17, 2025). To achieve maximum coverage of NF‐κB and the NF‐κB‐DNA complex during blind docking, only the grid box size was altered beyond the default values of the AutoDock software v. 4.2.6. Specifically, the number of points in *x*, *y*, and *z* dimensions was changed from the default value of 40 to the maximum value of 126. Spacing was kept at the default value of 0.375 Å and the grid box center coordinates were also kept at the default values, where *x* = 40.522, *y* = 16.84, and *z* = 36.636. The algorithm used for setting docking parameters was the Lamarckian algorithm. The default output of the algorithm for each ligand was the 10 most stable binding poses as judged by their binding energy values in kcal/mol.

### 4.8 Statistical Analysis

Data are presented as the mean ± standard error of the mean (SEM). Analyses were performed with Prism 8.0 software (GraphPad Software Inc., San Diego, CA, USA). Pretests were done to evaluate for normality and equality of variances. Statistical testing involved one‐way analysis of variance (ANOVA) followed by Tukey's post hoc comparison tests to determine significant differences between treatment groups. A *p*‐value of less than 0.05 (*p* < 0.05) was considered statistically significant.

## Funding

This research was funded by the University of Thessaly Financial and Secretarial Administration of research grants (SP, DEKA 5600.03.08.11).

## Conflicts of Interest

The authors declare no conflicts of interest.

## Data Availability

All the data are presented in the manuscript and raw data can be provided upon request.
